# Hyaluronic acid-FGF2-derived peptide bioconjugates for suppression of FGFR2 and AR simultaneously as an acne antagonist

**DOI:** 10.1186/s12951-023-01812-7

**Published:** 2023-02-17

**Authors:** Zijian Su, Yibo Zhang, Jieqiong Cao, Yuanmeng Sun, Yuling Cai, Bihui Zhang, Liu He, Zilei Zhang, Junye Xie, Qilin Meng, Lin Luo, Fu Li, Jingsheng Li, Jinting Zhang, Xiaojia Chen, An Hong

**Affiliations:** 1 Department of Cell Biology, College of Life Science and Technology, Jinan University; National Engineering Research Center of Genetic Medicine; Guangdong Provincial Key Laboratory of Bioengineering Medicine; Guangdong Provincial Biotechnology Drug & Engineering Technology Research Center, Jinan University, Guangzhou, 510632 Guangdong China; 2 The First Affiliated Hospital of Jinan University, Guangzhou, 510630 China

**Keywords:** HA polysaccharide, Oligopeptides, FGFR, AR, Signalling crosstalk, Acne therapy

## Abstract

Acne is a chronic skin condition that has serious consequences for mental and social well-being because it frequently occurs on the face. Several acne treatment approaches have commonly been used but have been hampered by side effects or weak activity. Thus, the investigation of the safety and efficacy of anti-acne compounds is of considerable medical importance. Herein, an endogenous peptide (P5) derived from fibroblast growth factors 2 (FGF2) was conjugated to the polysaccharide hyaluronic acid (HA) to generate the bioconjugate nanoparticle HA-P5, which suppresses fibroblast growth factor receptors (FGFRs) to significantly rehabilitate acne lesions and reduce sebum accumulation in vivo and in vitro. Moreover, our results show that HA-P5 inhibits both fibroblast growth factor receptor 2 (FGFR2) and androgen receptor (AR) signalling in SZ95 cells, reverses the acne-prone transcriptome, and decreases sebum secretion. Furthermore, the cosuppression mechanism revealed that HA-P5 blocks FGFR2 activation, as well as the YTH N6-methyladenosine RNA binding protein F3 (YTHDF3) downstream molecules, including an N6-methyladenosine (m6A) reader that facilitates AR translation. More importantly, a significant difference between HA-P5 and the commercial FGFR inhibitor AZD4547 is that HA-P5 does not trigger the overexpression of aldo-keto reductase family 1 member C3 (AKR1C3), which blocks acne treatment by catalyzing the synthesis of testosterone. Overall, we demonstrate that a polysaccharide-conjugated and naturally derived oligopeptide HA-P5 can alleviate acne and act as an optimal FGFR2 inhibitor and reveal that YTHDF3 plays a crucial role in signalling between FGFR2 and AR.

**Graphical Abstract:**

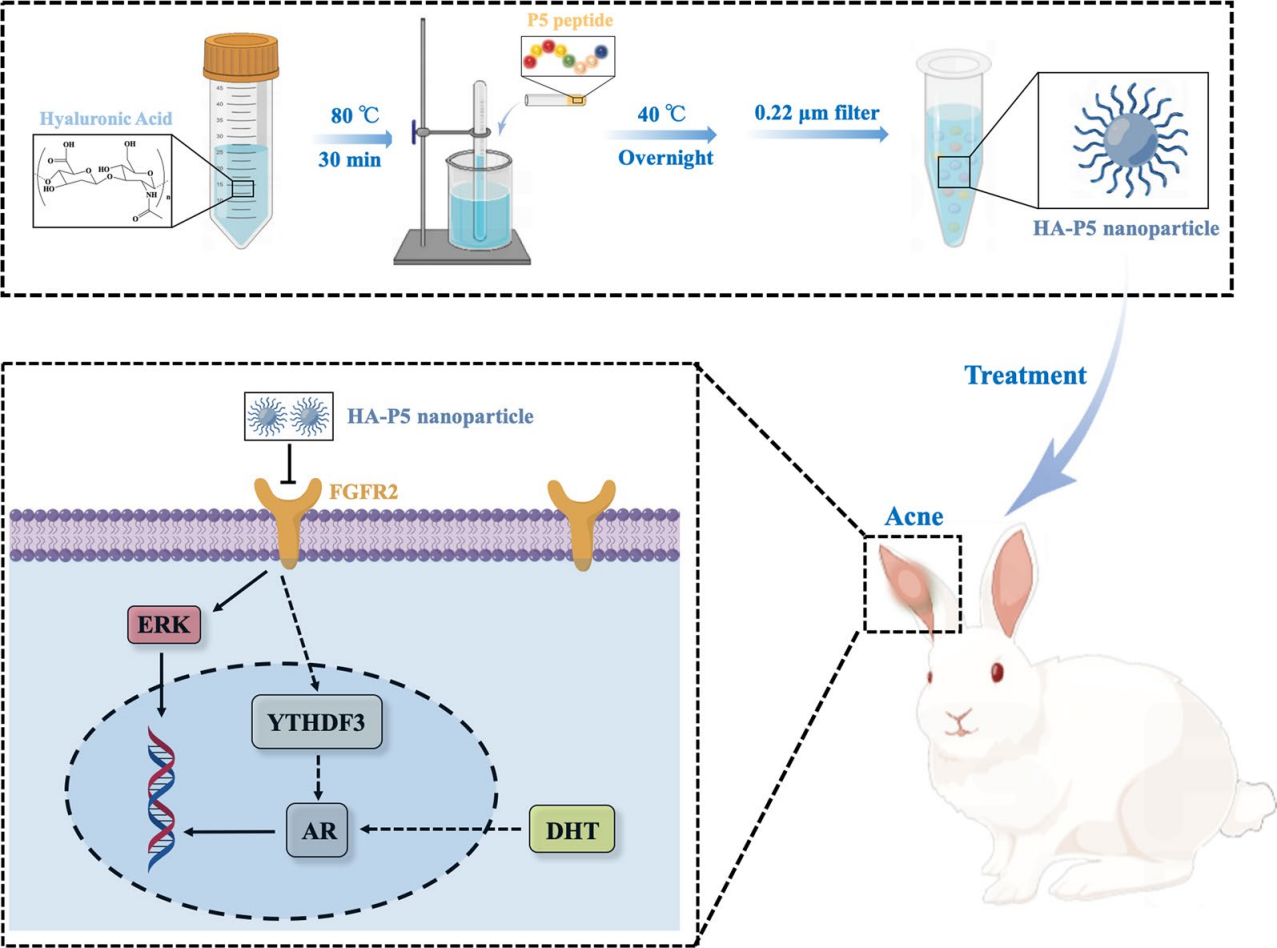

**Supplementary Information:**

The online version contains supplementary material available at 10.1186/s12951-023-01812-7.

## Introduction

Acne lesions are due to a chronic dermatological condition driven by multiple factors in the hair follicles and sebaceous glands. The lesions often occur in areas where sebaceous glands are intensively distributed, such as the face, which seriously affects one’s social well-being and mental health [1]. Several approaches have been applied in clinics against the pathologies of acne lesions [2–4]. For instance, retinoic acids were the first choice for mild acne [5–7]. Antiandrogen drugs were mainly prescribed for female patients, while glucocorticoids were considered for fulminant or aggregated acne [8]. However, these treatments are either time-consuming or have side effects, especially for pregnant women and other patients with special circumstances [9–11]. Therefore, investigations that clarify the mechanisms of the safety and efficacy of antiacne components with elucidated mechanisms have are of considerable medical interest.

Growth factors and their receptor signallings, such as fibroblast growth factors (FGFs) and their receptor signalling, contribute to the formation and development of acne lesions [12, 13]. As first reported in 1998, a patient with an FGFR2^S252W^ constituted activation mutant suffered from stubborn acne above his waist [14, 15]. Further studies subsequently demonstrated that FGFR2 mutants with constitutive activity contributed to systemic acne lesions in patients [16]. In skin tissues suffering from acne, AR signalling triggers the transcription of two FGFR2 ligands, FGF-7 (also known as keratinocyte growth factor, KGF) and FGF-10, which results in the activation of FGFR2 signalling [13, 17–21]. Therefore, FGFR2 signalling is closely related to the prognosis of acne therapy [22, 23]. Moreover, hyperactivated FGFR2 further drives the progression of acne lesions [24]. Taken together, these findings position as a potential therapeutic target for addressing acne lesions [25]. Given the roles of FGFs and FGFRs in the formation, development, and prognosis of acne lesions, we aimed to search for anti-acne candidates based on FGF/FGFR signaling Table 1.

**Table 1:** Comparison of different nanomaterials in acne treatment

Nanomaterials	Component	Advantage	Disadvantage
Liposomes	• Hydrophilic heads • Hydrophobic tails • Hydrophobic bilayer • Hydrophilic core	• Sustained drug release • Increase drug stability	• Low biodegradability • Low loading capacity • Gelation
Microspheres	• Hydrophilic phase • Hydrophobic phase • Cosurfactant • Surfactant	• High keratin penetration • Increase skin hydration	• Low biodegradability • Low loading capacity • Irritation and sensitizing potential of surfactants
Niosomes	• Antibody • Hydrophilic head • Hydrophobic tail • Hydrophobic drug	• Increase drug stability • Targeting	• Higher production cost • Lower-scale production
Solid nanoparticles	• Lipid matrix • Small molecule drug	• Increase drug stability • High keratin penetration	• Low biodegradability • Low loading capacity
HA-P5	• Peptide • Hyaluronic acid	• Large-scale production • Low production cost • Higher biocompatible • Higher biodegradability • High keratin penetration	• Instability

Currently, there are several FGFR inhibitors being explored in clinical and preclinical trials [26, 27]. These inhibitors can mainly be classified into synthetic chemicals targeted to the tyrosine kinase domain of FGFRs [28, 29] and biomacromolecules that neutralize FGFs or FGFRs, such as antibodies [30–32]. However, these exogenous inhibitors exhibit inevitable side effects [33, 34]. Hence, they have rarely been considered other than for the treatment of deadly diseases, such as malignancies [35, 36]. We have focused on investigating the roles of FGFs and FGFRs for years [37–41] and recently demonstrated that an endogenous FGFR2 inhibitory oligopeptide (P5), which is degraded from the basic fibroblast growth factor (bFGF, also known as FGF2) in vivo, effectively inhibits its downstream signalling. Importantly, P5 is a naturally occurring endogenous molecule that exhibits biological activities without terrible side effects [42]. This discovery of mild FGFR inhibitors with negligible toxicity will broaden the possible applications, especially for nonlethal inhibitors.

Recently, nanomaterials have been widely used in dermatopathy with three unique advantages: (1) they can improve the transdermal properties of active ingredients [43]; (2) they protect the stability of active ingredients (peptides/nucleic acids) in the skin microenvironment [44, 45]; and (3) prolong the retention time of active ingredients in the skin [46]. Typical nanomaterials for biomedical applications are polyvinyl alcohol (PVA), sodium alginate (SA), cyclodextrin (CD), HA, polyacrylic acid (PAA), polyacrylamide (PAAm), polycaprolactone (PCL), polyethylene glycol (PEG), and polylactic acid (PLA) [47]. In order to enhance the therapeutic efficacy, many new strategies were reported using graphene oxide, magnetic nanoparticles, and metal-organic frameworks as nanocarrier [48–50]. HA is one of the most widely used dressings in dermatological applications [51]. It has been successfully applied for the treatment of various acne scars [52–55]. We explored conjugating P5 with HA and synthesized HA-P5 nanoparticles. This study investigated the potential of HA-P5 for acne therapy, and the underlying mechanisms were investigated in vitro and in vivo.

## Materials and methods

### Synthesis and characterization of HA-P5

HA-P5 was synthesized by dissolving 5 mg of HA (10 kD) powder in 45 mL of Milli-Q water under magnetic stirring for 15 min at 80 °C when the solution turned transparent. EDC/NHS was dissolved in Milli-Q water and stirred for 30 min to get the EDC/NHS (20 mM/20 mM) solution. Next, the transparent HA solution was incubated with EDC/NHS solution at 30 °C for 60 min. Then, 5 mL P5 (1 mg/mL) was added to the reaction solution dropwise for 12 h at 40 °C. Finally, the solution was filtered with a 0.22 μm filter membrane to obtain the HA-P5 solution.

Transmission electron microscopy (TEM) samples were prepared by dispersing the pieces onto a holey carbon film on copper grids. The micrographs were obtained on a Tecnai G220 (Shimadzu, Japan) at 200 keV and Atomic Force Microscope (AFM) (Bioscope Catalyst/Multimode, USA). A dynamic light scattering (DLS) particle size analyser (Malvern 2000, USA) was used to determine the hydrodynamic diameters of the particles. The interaction between HA and P5 was detected by an infrared spectrometer (VERTEX70, USA) and zeta potential analyzer (Malvern 2000, USA). All measurements were performed at room temperature unless otherwise mentioned.

### Establishment of an acne model in the rabbit ear

Male New Zealand rabbits were purchased from Huadong Xinhua Experimental Animal Farm (China). Approximately 0.5 mL of 2% coal tar (Alfa Aesar #8007-45-2, Heysham, UK) was smeared within a range of 2 cm × 2 cm of the inner ear tube of New Zealand rabbits once every day for 2 weeks. Different treatments and controls were performed from the 15th day. Isotretinoin (Sigma‒Aldrich #4759-48-2, United States of America) and AZD4547 (MCE HY-13330, US) were dissolved in dimethyl sulfoxide (DMSO) (Sigma‒Aldrich #67-68-5, United States of America) and then diluted with the same HA polysaccharide. Different treatments (isotretinoin 0.1% (w/v); HA-P5 100 μM; AZD4547 5 μM) and controls (DMSO 0.1% (v/v); HA polysaccharide (10%) were administered daily for up to 20 days. Photographs were taken every 5 days until the 20 day, followed by execution and fixation (Sigma‒Aldrich, United States of America), and the area of effect was quantified as described in the reference [56]. All animal experiments were certified by the Experimental Animal Management Center of Jinan University and conducted following the “Guidelines for the Feeding, Management and Use of Laboratory Animals.”

### Preparation of paraffin sections

Firstly, the fixed rabbit ear tissue was dehydrated with gradient concentration ethanol for 30 min. Then the tissue was placed in xylene and soaked for 30 min twice and soaked in a mixture of paraffin: xylene = 1:1 solution for 1 h under 65 °C. After that, the tissues were soaked in melted paraffin wax twice for 60 min each time and then embedded. After the whole wax block is completely solidified, it is peeled off from the metal mold to get blocking embedded. Next, fixing the wax block on the microtome and smooth the wax block with a thickness of 4 μm. Spread the cut paraffin film flat in a 42 °C spreading machine. After the film was flattened in the water, the film was slowly pulled out from the bottom right below the film with a slide. Finally, absorbing the residual water around the slide with absorbent paper, place the slide in a 65 °C sheet oven for 40 min.

### Haematoxylin/eosin (H&E) staining

The paraffin sections were deparaffinized with xylene and rehydrated in a graded ethanol series. After rehydration, sections were soaked in water for 1 min, followed by haematoxylin staining for 15 min. Another round of soaking in running tap water was performed for 1 min, followed by quick washing in hydrochloric acid ethanol solution for 1–5 s. Scott blue liquid (Shyuanye # R20596-500 ml, Shanghai, China) was performed at the sections for 2 min followed by washing for 1 min. One minute of eosin staining was performed before the last round of washing. After that, ethanol gradient dehydration and transparentization were performed, and the section was sealed with the neutral resin.

### Fluorescence in situ hybridization experiment (FISH)

Dewaxing and hydration were performed for the paraffin sections, followed by incubation with 0.2 mol/L hydrochloric acid for 15 min and 0.5% Triton solution for 15 min. The sections were then raised with phosphate-buffered saline (PBS) in 0.01% diethylpyrocarbonate (DEPC) water for 5 min prior to treatment with 20 μg/mL proteinase K for 20 min. A 3% hydrogen peroxide solution was used to raise the section for 15 min, followed by washing with PBS (0.01% DEPC water). The diluted probe (Focofish, Guangzhou, China) and hybridization solution were mixed at a ratio of 1:50 as a working solution. Denaturation (85 °C for 3–5 min) and equilibration (37 °C for 2 min) were performed for the working solution prior to incubating the sections overnight at 37 °C. After incubation, the sections were rinsed with 2 ×saline sodium citrate (SSC) buffer and PBS three times. The sections were blocked in 3% Bovine serum albumin (BSA) for 30 min and incubated with the secondary antibody for 1 h at room temperature. Then, 0.15% hydrogen peroxide was used to prepare the tyramide signal amplification (TSA) dye before incubation with the sections in a 37 °C incubator for 15 min. The sections were rinsed with 2 ×SSC buffer 3 times and finally dried. 4,6-diamino-2-phenyl indole (DAPI) was used to stain the nucleus. Observation and image collection was performed under a fluorescence microscope (Olympus IX70, Japan).

### Cells and cell culture

SZ95 human sebaceous gland cells were from Dr.Zouboulis’s Lab originally [2], HSF human fibroblasts, and HaCaT human keratinocytes were purchased from NTCC (National Type Culture Collection, China) and maintained in dulbecco’s modified eagle medium (DMEM) (HyClone # SH30243.01, US) with 10% fetal bovine serum (FBS) (PAN # P30-3302, Germany) or 10% carbon-adsorbed serum (Certified Foetal Bovine Serum, BI, Israel). All cells were incubated at 37 °C in a 5% CO_2_ cell incubator (Bio-Rad GelDoc XR, US). Subculture of these cell lines was employed with 0.25% trypsin (Thermo Gibco #25200056, US).

### Cell proliferation assay

Cells were seeded in 96-well plates for 12 h prior to 0.5% FBS starvation for another 24 h to synchronize the cells. Both HA-P5 and AZD4547 were diluted with 0.5% FBS starvation medium before being added to the cells for 48 h. Cell Counting Kit-8 (CCK8) (Dojindo # CK04, Japan) reagent was added to the treated cells, and the 450 nm absorption was detected using a microplate reader (Tecan F50, Switzerland). The survival rate was calculated by dividing the A450 nm of the treated group by the A450 nm of the control group.

### Oil red O staining

The treated SZ95 cells in a 6-well plate were rinsed with PBS twice and fixed with 10% formaldehyde (Sigma‒Aldrich #F8775, United States of America) for 30 min before applying the oil red O working solution (Sigma‒Aldrich #1320–06-5, United States of America). The oil red O solution was then discarded after 15 min of staining and quickly rinsed with 60% isopropanol (Sigma‒Aldrich #67–63-0, United States of America), followed by PBS rinsing twice. The cells were then observed under an optical microscope (Olympus IX70, Japan).

### Triglyceride (TG) quantification

The treated SZ95 cells were rinsed with PBS twice, harvested in microtubes, and lysed on ice for 30 min. The lysates were then quantified by using a bicinchoninic acid (BCA) kit (Thermo Scientific 23225, US). The same cell lysate from each group was subjected to triglyceride quantification according to the standard protocol of the triglyceride test kit (Applygen E1013, China) by measuring the absorption at 550 nm under a microplate reader (Tecan F50, Switzerland).

### Cell transfection

Lipofectamine^™^ 3000 (7.5 μL, Thermo Scientific L3000075, US) was mixed with 5 μg target plasmid (HuiYuanYuan, China) or 75 pmol target siRNA (Ribobio, China), diluted with 250 μL serum-free DMEM and incubated at room temperature for 15 min as working solutions. Cells were rinsed twice with PBS and incubated with serum-free DMEM before adding the working solutions, followed by a six-hour incubation in a 37 °C, 5% CO_2_ constant temperature incubator (RSBiotech Galaxy R + , US). The medium was then replaced with DMEM containing 10% FBS for another 48 h of incubation prior to further experiments.

### RNA extraction and reverse transcription

Treated cells were harvested with RNAios Plus (Takara #9109, Japan) and extracted with an equal volume of chloroform. After centrifugation at 4 °C and 12,000 × g for 15 min, the top supernatant layer was carefully harvested and mixed with the same volume of isopropanol. After 2 h freezing, the samples were then centrifuged at 4 °C and 12000 × g for 10 min. The precipitates were then rinsed with 75% ethanol in DEPC water (Solarbio R1600, China) and air dried. The precipitated RNA was then dissolved in DEPC water (Solarbio R1600, China) and quantified with a Nanodrop (Thermo Scientific NanoDrop 8000, USA). Reverse transcription was performed using the PrimeScript IV 1st strand cDNA synthesis kit (TaKaRa #6215, Japan) according to the standard protocol.

### Quantitative polymerase chain reaction (qPCR)

The cDNA samples were mixed with 2X qPCR master mix (MCE HY-K0501, US) and PCR primer pairs. Samples were amplified using 95 °C denaturation for 30 s, 60 °C annealing, and extension for 60 s, 40 cycles in total, in a real-time PCR machine (Bio-rad CFX96, US). The expression of AR, YTHDF3, and AKR1C3 was presented as the relative expression level by using the housekeeping gene β-actin as a control. The sequence of the primers was listed as follows:

**Table:** 

AR	Forward 5′-GTACAGCCAGTGTGTCCGAA-3′
	Reverse 5′-TTGGTGAGCTGGTAGAAGCG-3′
YTHDF3	Forward 5′-CATTGTGGACCCGAGAAGCA-3′
	Reverse 5′-GACATTCTTCACCGCAACCC-3′
AKR1C3	Forward 5′-AGGAATGGATTCCAAACACCA-3′
	Reverse 5′- GGCGGAACCCAGCTTCTATT-3′
β-Actin	Forward 5′-GTCATTCCAAATATGAGATGCGT-3′
	Reverse 5′-GCTATCACCTCCCCTGTGTG -3′

### RNA-seq

Total RNA was extracted and sent to Beijing Biomark Biotechnology Co., Ltd. on dry ice for library establishment and sequencing. Data analysis and mapping were performed through the company's official platform server or the DAVID bioinformatics databases. The charts were drawn using RStudio software.

### Protein extraction and quantification

Treated cells were lysed using RIPA Lysis Solution (Thermo 89900, US) and centrifuged at 10000×*g*. The supernatant was collected and quantified using a BCA Kit (Thermo Scientific 23225, US).

### Western blotting

The protein was mixed with 2X Laemmli loading buffer (Sigma S3401, US) and incubated at 95 °C for 5 min. The sample was loaded to a 12% SDS-polyacrylamide gel for electrophoresis and transferred to a PVDF membrane (Millipore ISEQ00010, Germany). After blocking with skimmed milk powder (Bio basic #A600669-0250, Canada) or 5% BSA (Sigma‒Aldrich#A8806-5G, United States of America), the membrane was then incubated with primary antibodies (Cell Signalling Technology #19672; #3471; #4691; #4060; #4370; #4695; #5174; #3700, US; Abcam #ab209899, UK) overnight at4 °C. The incubated membrane was washed with Tris-Buffered Saline and Tween 20 (TBST) three times (5 min/time) before incubation with the secondary antibody (Cell Signalling Technology #7074; #7076, US) for another hour at room temperature. After that, the membrane was washed with TBST three times prior to being developed with enhanced chemiluminescence (ECL) (Abbkine # K22030, US).

### Statistical analysis

All the data were analyzed by GraphPad Prism software and are presented as the mean ± SD. One-way ANOVA or two-way ANOVA was used for multiple comparisons, while Student's t-test was used for comparing two groups.

## Results

### Synthesis and characterization of HA-P5

**Fig. 1:**
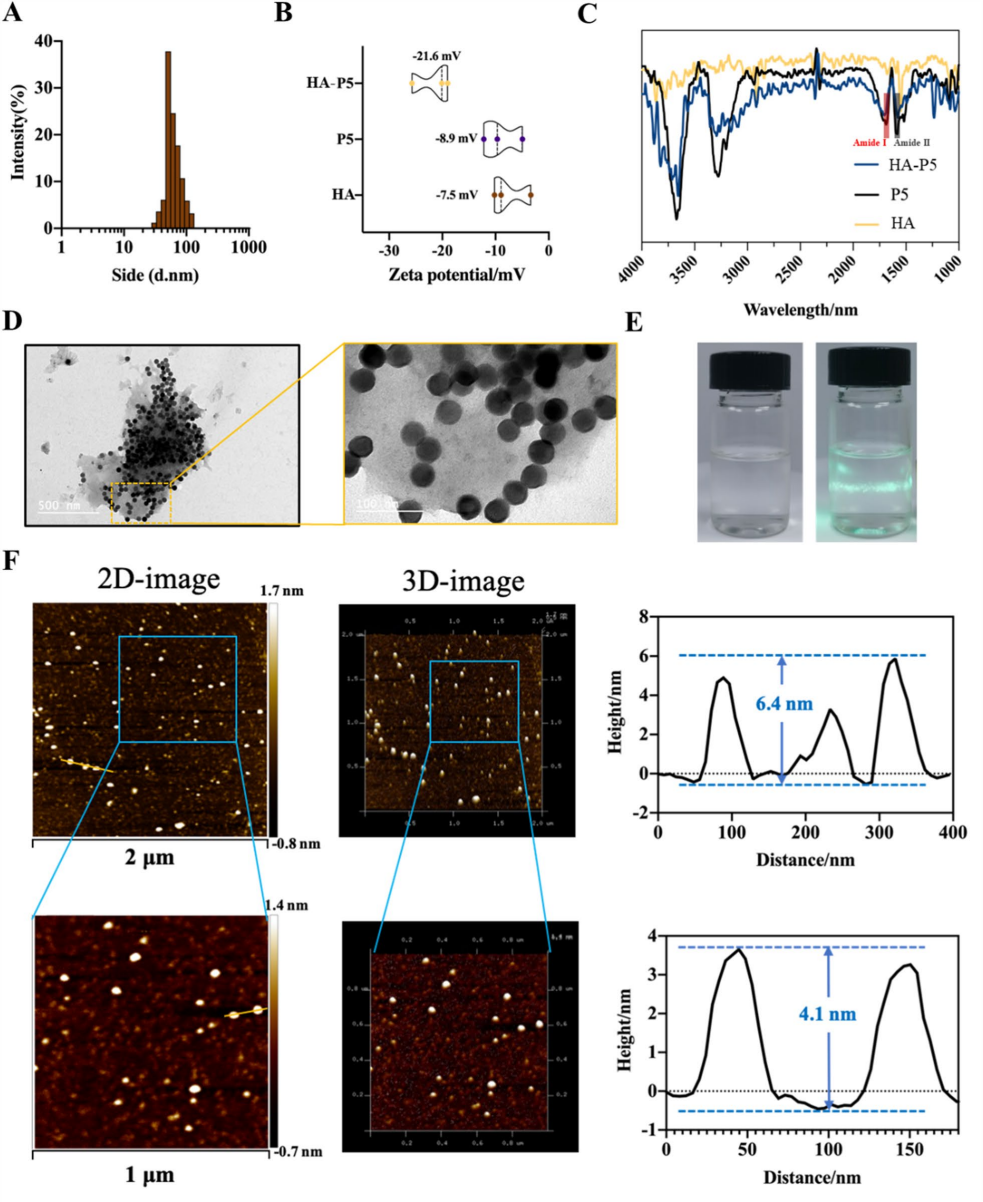
Preparation and characterization of HA-P5. **A** Particle size distribution of the HA-P5; **B** Zeta potential analysis of P5, HA and HA-P5; **C** Infrared spectrometer analysis of P5, HA and HA-P5; **D** TEM image of HA-P5; **E** The dundal effect of HA-P5; **F** AFM image and particle size data analysis of HA-P5

HA-P5 was synthesized by heating HA (3 kD) to 80 °C in Milli-Q water for 30 min, followed by adding P5 peptide (1 mg/mL) for 12 h at 40 °C. DLS (Fig. 1A) measurement for the HA-P5 solution demonstrates an average diameter of approximately 67 nm. Typical transmission electron microscopy (Fig. 1D) and atomic force microscopy (Fig. 1F) images showed that HA-P5 is a spherical structure with a diameter of approximately 40–80 nm, which is consistent with the DLS results. Zeta potential and infrared spectroscopy were applied to verify the interaction between HA and the P5 peptide. The zeta potential (Fig. 1B) of HA-P5 (− 21.6 mV) was higher than that of HA (− 7.5 mV) and P5 (− 8.9 mV), which might indicate the interaction between HA and P5. The infrared spectra (Fig. 1C) illustrate that the peaks present in the HA-P5 bioconjugates included the peaks characteristic of HA and P5, and the amide I and amide II peaks in samples of HA-P5 (amide I: 1686 cm^−1^, amide II: 1522 cm^−1^) and P5 (amide I: 1686 cm^−1^, amide II: 1572 cm^−1^). Moreover, the –OH peak (3674.18 cm^−1^) in HA-P5 was blue-shifted compared with that in P5 (3718.54 cm^−1^). Our results indicated that nanospherical HA-P5 with a diameter of approximately 70 nm was successfully synthesized.

### HA-P5 remedies acne symptoms in vivo

To examine the therapeutic activity of HA-P5, the Kligman method [57], which has been commonly used internationally, was employed to establish an acne model in rabbits (Additional file 1: Figure S1). As shown in Additional file 1: Figure S2, S3, rabbit ears that were exposed to coal tar demonstrated a coarser epidermis, larger hair follicles, and thicker stratum corneum. The hair follicles on the rabbit ear epidermis became abnormally expanded, with acne-like horn plugs and desquamation. Isotretinoin, a clinical antiacne agent, was introduced as a control. Isotretinoin treatment significantly improved the health of rabbit ears, resulting in less keratinization and smaller hair follicles. These results indicated that the rabbit acne model was successfully established.

**Fig. 2:**
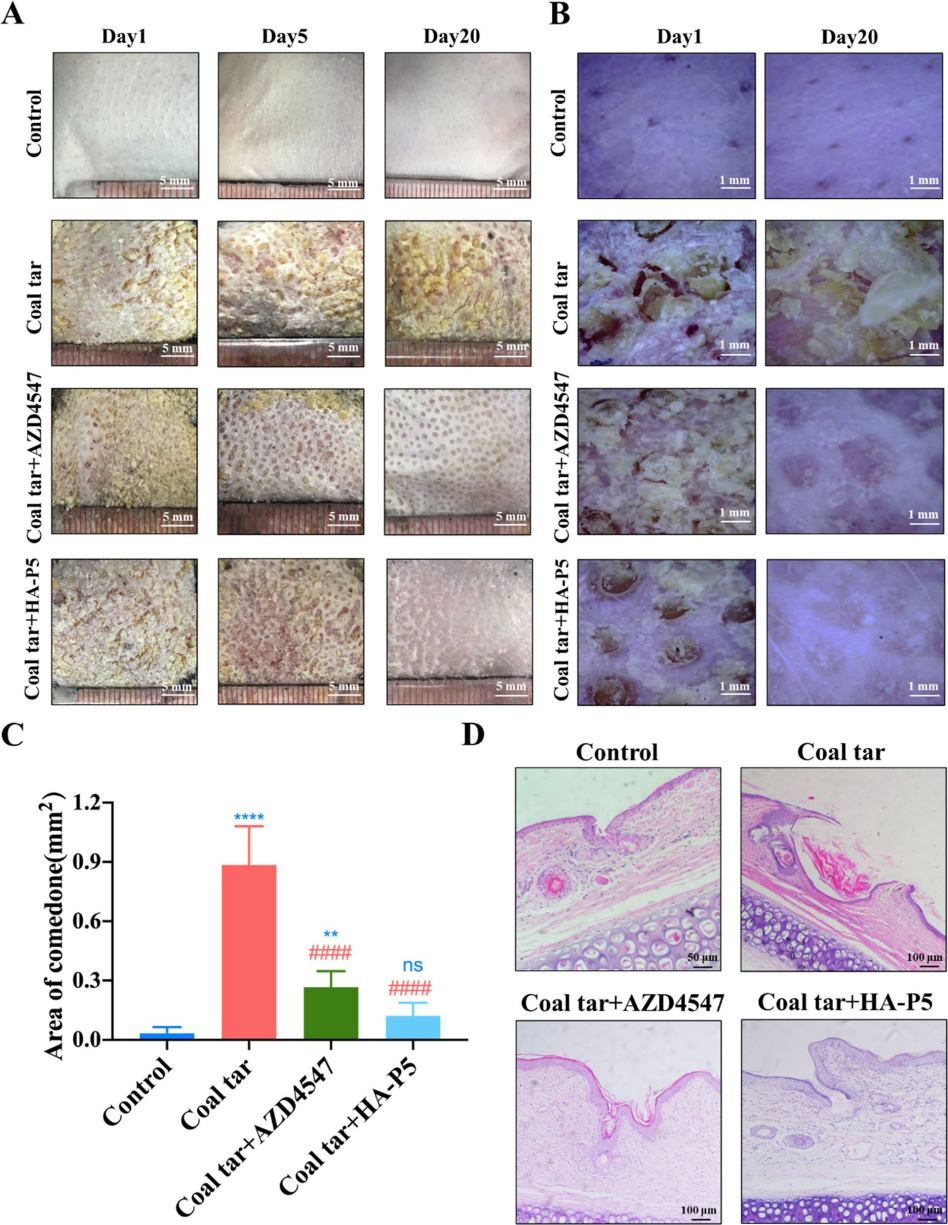
HA-P5 remedied acne lesions in vivo. **A** HA-P5 and AZD4547 remedied acne on the ears of male New Zealand rabbits with smoother epidermis and thinner stratum corneum; **B** HA-P5 and AZD4547 diminished the hair follicles on the skin of rabbit ears; **C** Quantification of the area of comedones the rabbit ears. One-way ANOVA was employed for statistical analysis. Ns in blue refers to p > 0.05 compared to the control group ** in blue color refers to p < 0.01 compared to the control group, and **** in blue color refers to p < 0.0001 compared to the control group. #### in red refers to p < 0.0001 compared to the coal tar group; **D** Typical H&E staining images of the vertical sections of the hair follicles. n = 3

The local application of HA-P5 demonstrated a time-dependent therapeutic effect on acne, which was indicated by a smoother epidermis, a thinner stratum corneum, and tighter hair follicles (Fig. 2A, B and Additional file 1: Figure S2). A pan FGFR inhibitor, AZD4547, produced a similar overall effect as HA-P5; however, the effect was weaker than with HA-P5 (Fig. 2A, B). Additionally, we quantified the area of the comedones, and the results demonstrated that coal tar significantly increased the area of the comedones and that this could be reversed by both HA-P5 and AZD4547 (Fig. 2C). Solvent controls, including HA and DMSO, demonstrated no significant treatment effect on the rabbit ears (Additional file 1: Figure S2, S3). Moreover, H&E staining of the skin sections indicated that both HA-P5 and AZD4547, similar to isotretinoin, reversed the damage to the skin and follicle hairs induced by coal tar (Fig. 2D). These results collectively indicate that HA-P5 remedies acne as an FGFR inhibitor.

### HA-P5 and AZD4547 reverse the acne-prone transcriptome induced by dihydrotestosterone (DHT) in SZ95 sebaceous cells

**Fig. 3:**
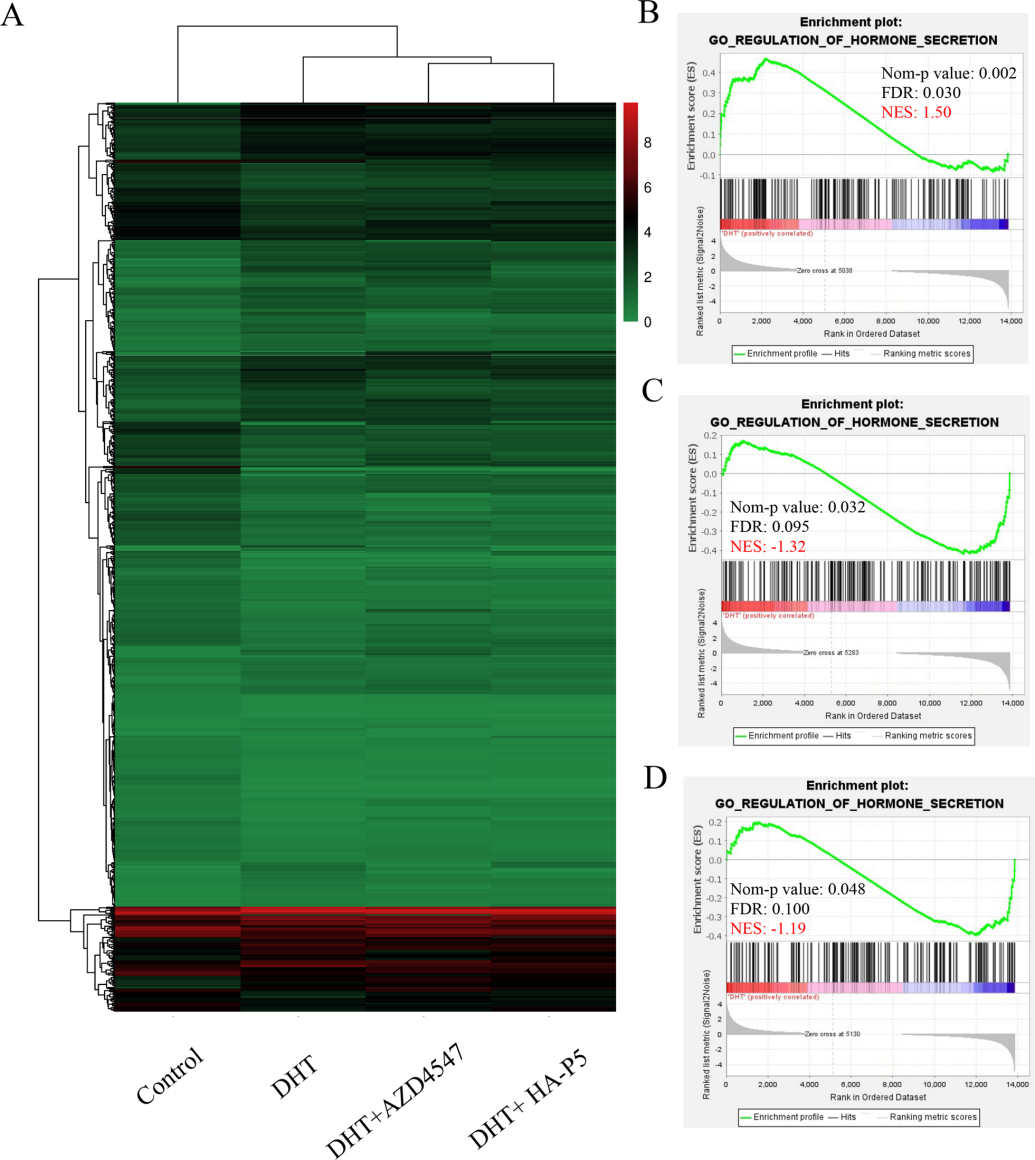
HA-P5 reversed the acne-prone transcriptome induced by DHT in SZ95 sebaceous gland cells. **A** Heatmap indicates the differential expressed genes induced by HA-P5 and AZD4547 with the presence of DHT; **B** DHT stimulated the expression of hormone section-related genes; **C** AZD4547 inhibited the expression of hormone section-related genes induced by DHT; **D** HA-P5 inhibited the expression of hormone section-related genes induced by DHT

Since sebaceous gland cells are pivotal for the formation and development of acne [58, 59], the SZ95 human sebaceous gland cell line was employed to investigate the potential mechanisms by which HA-P5 remedies acne as an FGFR inhibitor. DHT was chosen to mimic the stimulation of androgen on the hair follicles, while HA-P5 and AZD4547 were applied in DHT-pretreated SZ95 cells. The transcriptomes of SZ95 cells with different treatments were analysed using next-generation sequencing. Figure 3A demonstrates the differentially expressed genes (DEGs) in SZ95 cells with different treatments. We further performed gene set enrichment analysis (GSEA) for these EDGs.

Interestingly, a set of genes related to the regulation of hormone secretion, which is pivotal for the formation and development of acne, significantly changed across these treatment groups. In SZ95 cells, DHT stimulation overall elevated the regulation of hormone secretion-related genes, with a normalized enrichment score (NES) of 1.50 (Fig. 3B). Moreover, the presence of either HA-P5 or AZD4547 successfully abolished the stimulatory effect of DHT, indicated by NESs of − 1.19 and 1.32, respectively (Fig. 3C, D). These results suggested the involvement of hormone secretion in the antiacne activities of HA-P5 as an FGFR inhibitor.

### HA-P5 inhibits the proliferation of keratinocytes and reduces sebum accumulation in sebaceous gland cells

**Fig. 4:**
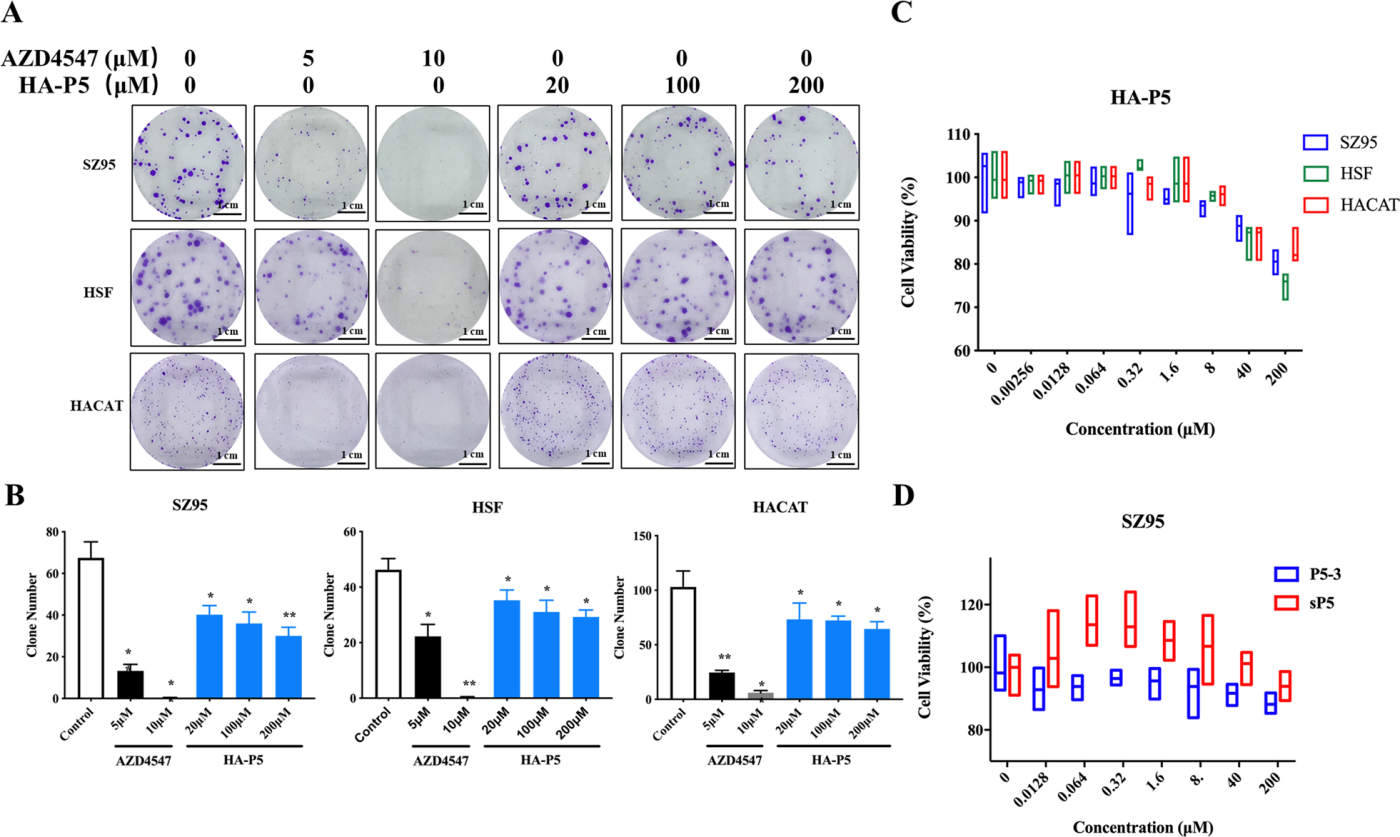
HA-P5 or AZD4547 inhibits the proliferation of SZ95 human sebaceous gland cells, HaCaT human keratinocytes, and HSF human fibroblasts. **A** HA-P5 and AZD4547 inhibit the colony formation in SZ95, HaCaT, and HSF cells; **B** Quantification of the inhibitory effect of HA-P5 and AZD4547 on the colony formation in SZ95, HaCaT, and HSF cells. One-way ANOVA was employed for statistical analysis. *p < 0.05, **p < 0.01, ***p < 0.001, ****p < 0.0001; **C** HA-P5 and AZD4547 inhibits the proliferation of SZ95, HaCaT, and HSF cells; **D** The cell proliferation inhibitory effect of HA-P5-3 and HA-sP5 peptides

Considering the significant changes in gene sets related to the regulation of hormone secretion, which is closely related to cell proliferation and sebum secretion during the formation and development of acne, sebaceous gland cells, fibroblasts, and keratinocytes were employed to examine the antiproliferative activity of HA-P5. In these three DHT pretreated cell lines, HA-P5 or AZD4547 significantly inhibited clonal formation and proliferation in a dose-dependent manner (Fig. 4A–C).

To verify the importance of the HA-P5 sequence, we substituted two crucial amino acids (Q4 and E6) and synthesized a P5-3 peptide and an sP5 peptide with the reverse sequence to synthesize HA-P5-3 and HA-sP5. The results demonstrated that the proliferation inhibitory effect of HA-P5 on SZ95 cells became insignificant after substitution or reversion (Fig. 4D), indicating the crucial role of the sequence of HA-P5.

**Fig. 5:**
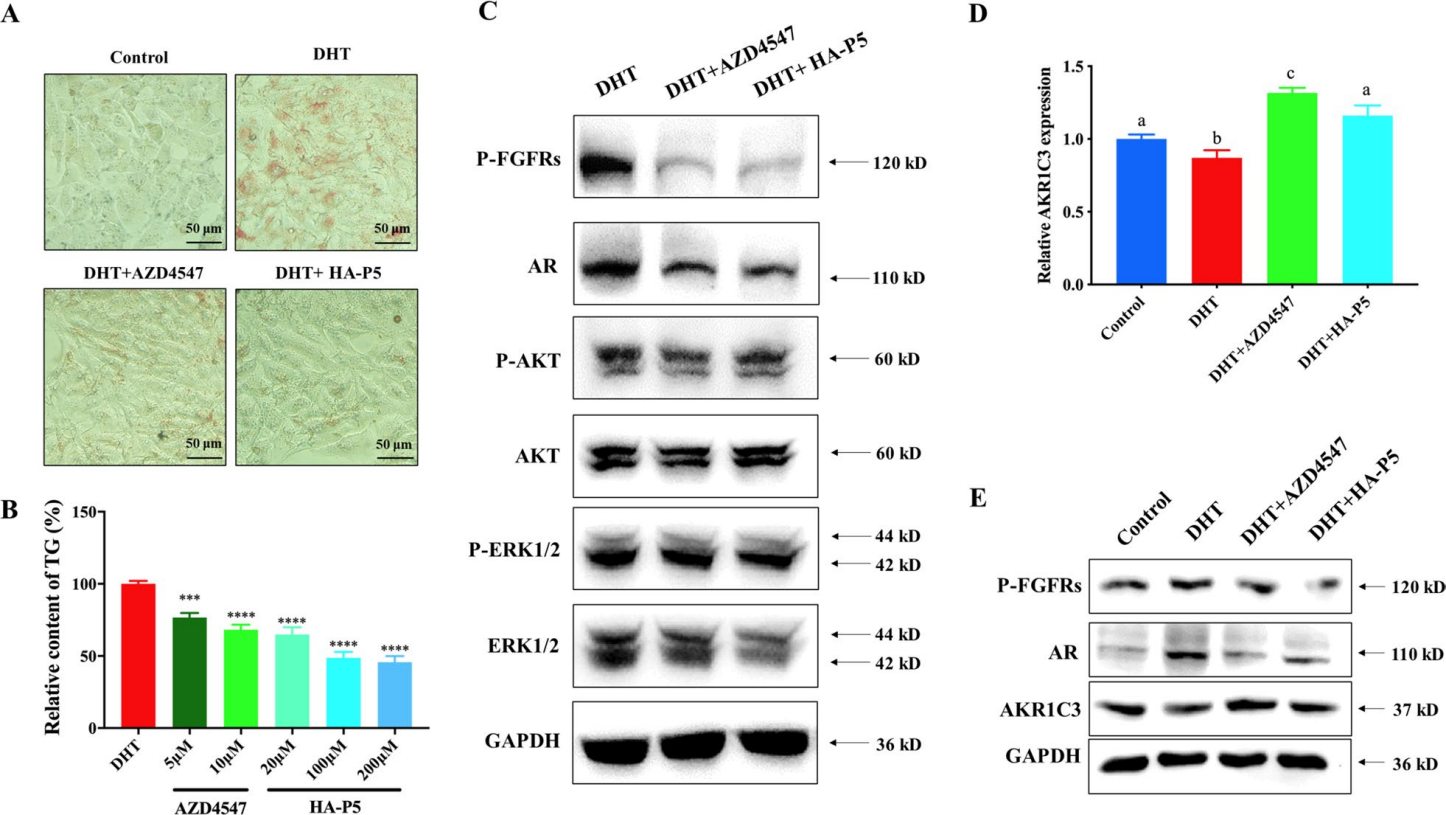
HA-P5 reduced the sebum accumulation in SZ95 cells with the involvement of FGFRs and AR signalling pathways. **A** Oil red O staining indicates HA-P5 and AZD4547 reduced the sebum over-accumulation induced by DHT in SZ95 cells; **B** Quantification of TG in SZ95 cells treated with HA-P5 and AZD4547 with the presence of DHT. One-way ANOVA was employed for statistical analysis. ***p < 0.001, ****p < 0.0001; **C** Western blotting analyzes the key molecules of FGFRs and AR signalling pathways in response to the treatment of HA-P5 and AZD4547 with the presence of DHT; **D** qPCR quantified the expression of AKR1C3 in SZ95 cells treated with HA-P5 and AZD4547 with the presence of DHT. One-way ANOVA was employed for statistical analysis. Columns with different letters refer to p < 0.01; **E** Western blotting analyzes the protein level of AKR1C3 in SZ95 cells treated with HA-P5 and AZD4547 with the presence of DHT

Considering that excessive accumulation of sebum contributes to the formation and development of acne [60], we evaluated the regulatory activities of HA-P5 on sebum formation in SZ95 cells. Excessive sebum formation was observed in SZ95 cells treated with DHT, while it was significantly reversed by either HA-P5 or AZD4547 (Fig. 5A). The inhibitory effect was further quantified by measuring TG (Fig. 5B), indicating the dose-dependent inhibitory activities of HA-P5 on sebum formation in sebaceous gland cells as an FGFR inhibitor.

### HA-P5 reduces proliferation and sebum accumulation in SZ95 cells by inactivating FGFRs and AR signalling

To investigate how HA-P5 reduces sebum accumulation, we measured the activation of FGFRs and AR and their downstream kinases using western blotting. As shown in Fig. 5C, both HA-P5 and AZD4547 significantly inhibited the phosphorylation of FGFRs. They reduced the expression of AR, as well as their downstream kinases, AKT and ERK, indicating that HA-P5 and AZD4547 significantly inhibit FGFRs and AR signalling in SZ95 cells. These results collectively demonstrated that HA-P5 and AZD4547 might reduce sebum accumulation with the involvement of FGFRs and AR inactivation in SZ95 human sebaceous gland cells.

**Fig. 6:**
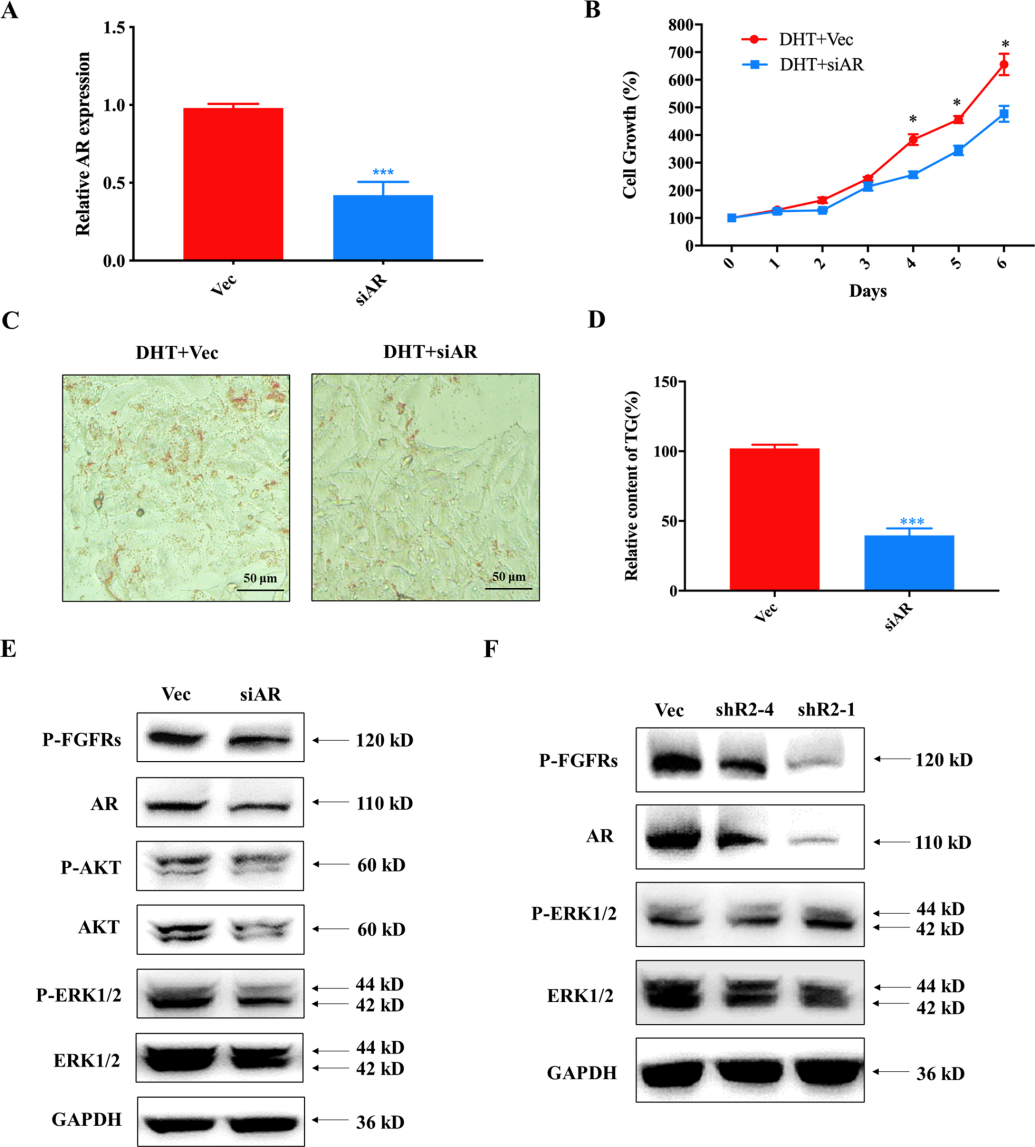
Crosstalk between FGFRs and AR signalling during the formation of acne. **A** Quantification of AR transcriptional level in SZ95 cells treated with AR siRNA. One-way ANOVA was employed for statistical analysis. ***p < 0.001; **B** Growth inhibition in SZ95 cells induced by AR siRNA. One-way ANOVA was employed for statistical analysis. *p < 0.05; **C** Oil red O staining indicates that knockdown of AR reduced the sebum accumulation in SZ95 cells; **D** Quantification of TG in SZ95 cells treated with AR siRNA. One-way ANOVA was employed for statistical analysis. ***p < 0.001; **E** Western blotting analyzes the key molecules of FGFRs and AR signalling pathways in response to the knockdown of AR in SZ95 cells; **F** Western blotting analyzes the key molecules of FGFRs and AR signalling pathways in response to knockdown of FGFR2 in SZ95 cells

Extensive studies have shown that AR signalling stimulates sebum secretion in hair follicles and drives the formation and development of acne [61]. Small interfering RNA (siRNA) was employed to knock down AR in SZ95 cells to validate the role of AR signalling in regulating proliferation and sebum accumulation in human sebaceous gland cells. AR knockdown was indicated by the significantly reduced mRNA level (Fig. 6A), which decreased cell proliferation and sebum formation in SZ95 sebaceous gland cells (Fig. 6B–D). These results suggested that AR might drive acne by stimulating proliferation and sebum formation in sebaceous gland cells.

Considering that the targets of both HA-P5 and AZD4547 are FGFRs, we attempted to investigate the role of FGFRs in regulating proliferation and sebum formation in sebaceous gland cells, as well as the internal relationship between FGFRs and AR signalling. Figure 6E demonstrates that AR contributed to the activation of FGFRs and downstream kinases, such as AKT and ERK. Moreover, short hairpin RNA (shRNA) was used to knock down FGFR2, a major target of HA-P5. The results demonstrated that the AR protein level dropped due to the loss of FGFR2, indicating positive feedback between FGFRs and AR signalling (Fig. 6F). These results collectively suggested that HA-P5 and AZD4547 might block the activation of FGFR2 to reduce the levels of the AR protein, which is pivotal for the proliferation and accumulation of sebum in sebaceous gland cells.

### FGFR2 signalling facilities the translation of AR in a YTHDF3-dependent manner

It has been extensively reported that transcription factors, such as p53, GATA binding protein 6 (GATA6), and forkhead box O1 (FOXO1), regulate the expression of AR. To further investigate how FGFR2 signalling affects AR signalling, we analyzed the transcriptome and found that the expression of p53, GATA6, and FOXO1 remained unchanged. The substrates of these transcription factors, including p21, platelet-derived growth factor subunit B (PDGFB), and peroxisome proliferator activated receptor gamma (PPARG), were also unchanged, suggesting that these transcription factors might not contribute to the regulation of AR expression (data not shown). Interestingly, we noticed that the AR transcription did not demonstrate significant changes after exposure to FGFR2 inhibitors (HA-P5 and AZD4547). However, the AR protein level decreased dramatically in the presence of either HA-P5 or AZD4547 (Fig. 5C, E).

**Fig. 7:**
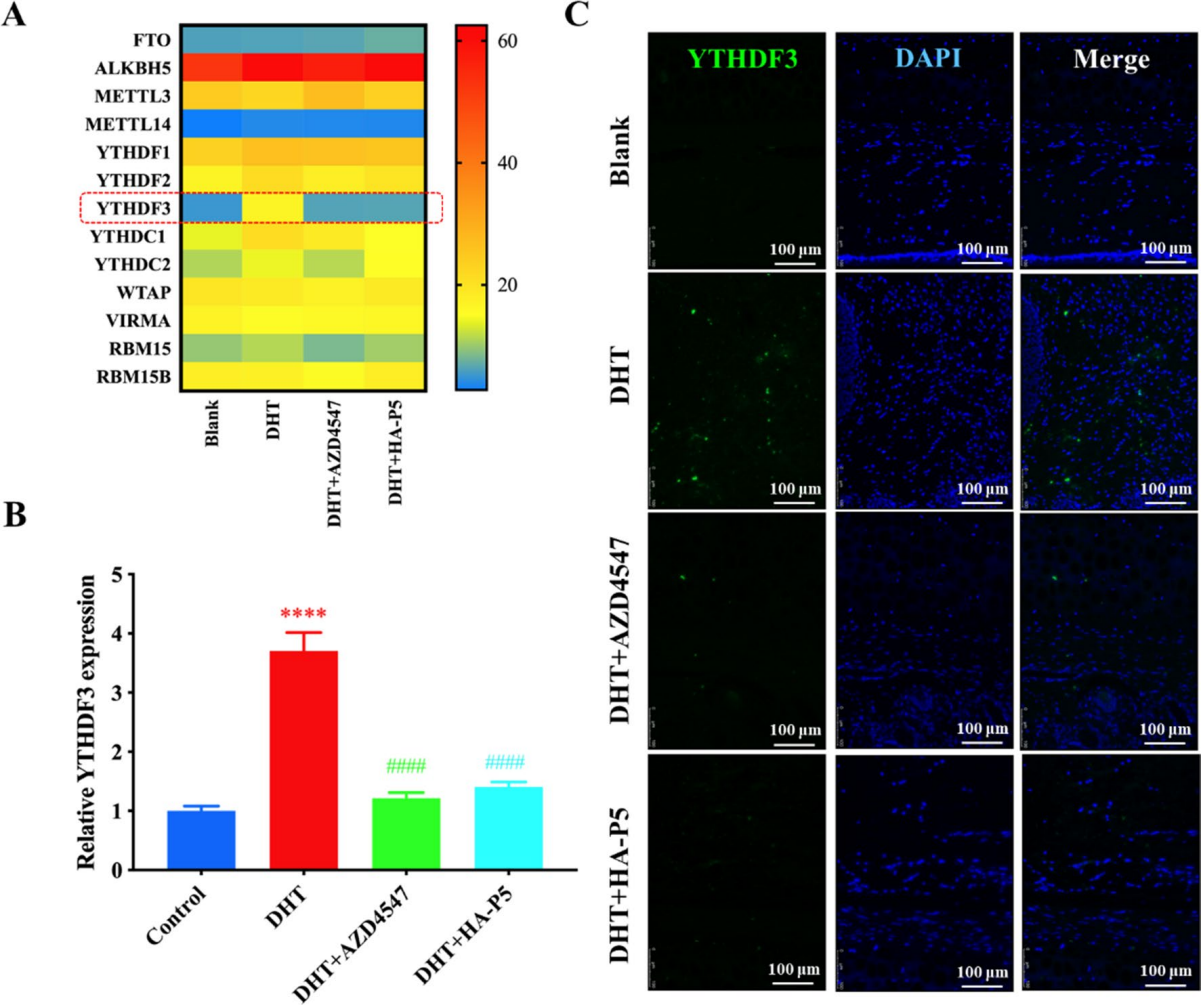
HA-P5 inhibited FGFR to reverse the overexpression of YTHDF3 induced by DHT. **A** Heatmap indicates the expression of m6A-related genes in SZ95 cells treated with HA-P5 and AZD4547 with DHT; **B** qPCR quantified the expression of YTHDF3 in SZ95 cells treated with HA-P5 and AZD4547 with the presence of DHT. One-way ANOVA was employed for statistical analysis. **** refers to p < 0.0001 compared to the control group, while #### refers to p < 0.0001 compared to the DHT-treated group; **C** Typical FISH images of the transcription of YTHDF3 in rabbit ears treated with HA-P5 and AZD4547 with the presence of DHT

Considering the conformity of the mRNA and AR protein levels, we hypothesized that FGFR2 signalling regulates AR at a posttranscriptional level. To validate this hypothesis, we first aimed to analyze the transcription of m6A-related genes, which regulate the posttranscriptional modification of RNA, in SZ95 cells and unexpectedly found that the expression of an m6A reader, YTHDF3, was elevated in the presence of DHT. This elevation of the YTHDF3 transcript was then quelled to the negative control level in response to either AZD4547 or HA-P5 treatment. Other m6A-related genes remained unchanged in all these groups (Fig. 7A). Moreover, the expression of YTHDF3 was verified by employing qPCR (Fig. 7B). FISH was employed to measure the transcript of YTHDF3 in rabbit ears because there are no primary commercial antibodies available for rabbit YTHDF3. As shown in the typical FISH images, fluorescent signalling increased in response to coal tar treatment, which drives the formation of acne. Similarly, the YTHDF3 transcript became undetectable when the rabbit ear was exposed to either P5 or AZD4547 (Fig. 7C). These results suggested that YTHDF3 might be a candidate that mediates the crosstalk between FGFR2 and AR signalling in vivo and in vitro.

**Fig. 8:**
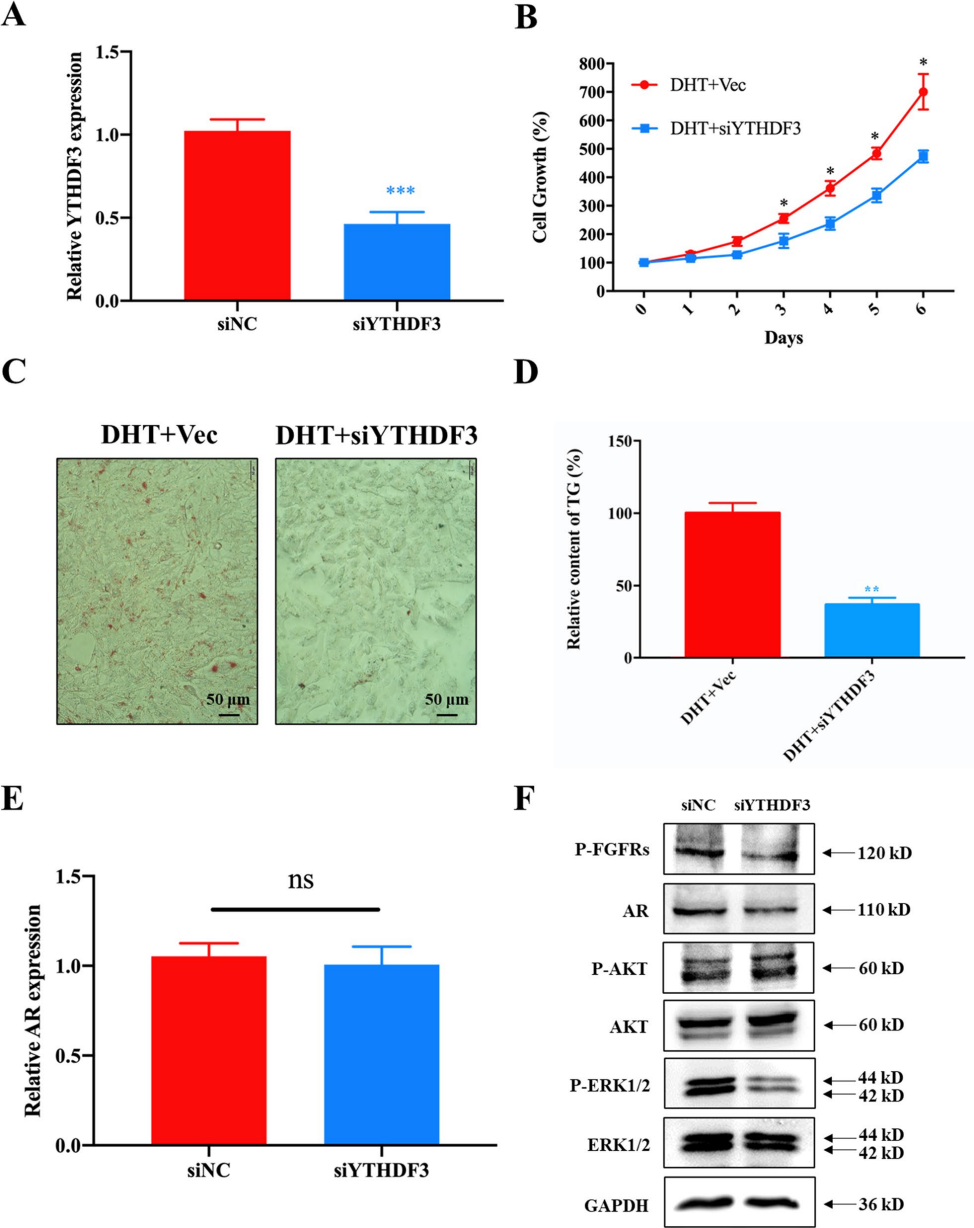
HA-P5 and AZD4547 inhibited the AR translation in a YTHDF3-dependent manner in SZ95 cells. **A** qPCR quantified the expression of YTHDF3 in SZ95 cells treated with YTHDF3 siRNA. One-way ANOVA was employed for statistical analysis. ***p < 0.001; **B** Growth inhibition in SZ95 cells in response to knockdown of YTHDF3. One-way ANOVA was employed for statistical analysis. *p < 0.05; **C** Knockdown of YTHDF3 reduced the sebum accumulation in SZ95 cells as indicated by typical Oil red O staining images; **D** Quantification of TG in SZ95 cells treated with YTHDF3 siRNA. One-way ANOVA was employed for statistical analysis. **p < 0.01; **E** qPCR quantified the transcription of AR in SZ95 cells treated with YTHDF3 siRNA; **F** Western blotting analyzes the translation of AR protein in response to knockdown of YTHDF3 in SZ95 cells

To verify the importance of YTHDF3 in the connection of FGFR2 and AR signalling, as well as its role in stimulating the formation and development of acne, siRNA was employed to knock down YTHDF3 in SZ95 cells. As shown in Fig. 8A, over half of the YTHDF3 mRNA was depleted with siYTHDF3. In DHT-treated SZ95 cells, cell proliferation and sebum accumulation significantly decreased when YTHDF3 was knocked down (Fig. 8B–D), suggesting that DHT-induced SZ95 cell proliferation and sebum accumulation are YTHDF3 dependent. Moreover, AR was quantified at both the transcriptional and translational levels. The results demonstrated that the AR protein level decreased significantly after the knockdown of YTHDF3, accompanied by the inactivation of AKT and ERK. In contrast, the AR mRNA level remained unchanged (Fig. 8E, F). Collectively, these results indicated the pivotal role of FGFR2 in connecting FGFR2 and AR signalling and therefore mediating the antiacne activities of FGFR inhibitors.

### HA-P5 does not trigger antagonism by androgen

Interestingly, we noticed that the expression of AKR1C3 (encoding 17β-HSD5, an enzyme that catalyzes testosterone synthesis) significantly increased with AZD4547. However, treatment of SZ95 cells with HA-P5 did not trigger the expression of AKR1C3 (Fig. 5D). Western blotting was also employed to verify the changes in AKR1C3 at the protein level, and the results demonstrated an increase in AKR1C3 protein in SZ95 cells treated with AZD4547 but not HA-P5 (Fig. 5E). Since AKR1C3 encodes an enzyme for the synthesis of androgen [62], the elevation of AKR1C3 enhances AR signalling, which is not conducive to treating acne. These results indicated that HA-P5 might exhibit better antiacne activities than other FGFR inhibitors, such as AZD4547.

## Discussion

Acne is a chronic skin disease that seriously affects social well-being and mental health. It is driven by excessive sebaceous secretion and keratinization in the hair follicle sebaceous gland [63]. Although several approaches have been applied in the clinic, there is still a lack of effective and safe agents for acne therapy. In this study, we highlighted the HA polysaccharide conjugated and naturally derived oligopeptide HA-P5, which demonstrated antiacne activities in vivo and in vitro by inhibiting FGFR2/YTHDF3/AR signalling.

For many years, the traditional treatment of acne has used different antibiotics, acids, benzoyl peroxide, and retinoids [64]. However, there are some side effects with these molecule drugs, such as peeling, redness, dryness, and gastrointestinal upset [65]. To address these side effects, nanotechnology was applied in acne treatment. Liposomes [66], microspheres [67], niosomes [68], and solid nanoparticles [69] were as nanocarriers for the treatment of acne. As shown in Table 1. Compared to these nanomaterials, HA-P5 has two unique advantanges: (1) Compared to metal nanoparticles, such as gold/silver nanoparticles, HA-P5 is mainly composed of peptides and hyaluronan, which are biodegradable in vivo with higher biosafety; (2) The synthesis process of HA-P5 is simpler and lower cost than lipid nanocarriers. Taken together, HA-P5 is a novel nanomaterial with broad application prospects for acne treatment.

Among several pathologies of acne lesions, amplification of AR signalling is vital. Current knowledge indicates that AR drives the transcription of FGF7/FGF10, further facilitating the proliferation of sebaceous gland cells and keratinocytes [25]. In this manner, FGFR signalling is one of the downstream effects of AR signalling during the formation of acne lesions. In this study, inhibition of FGFRs by HA-P5 and AZD4547 significantly abolished the acne-stimulating effect of DHT and coal tar in vitro and in vivo, indicating the importance of FGFR signalling in acne lesions. Previously, the roles of FGFs and FGFRs in acne lesions have been addressed [22], and our results are consistent with the findings of this previous study. Moreover, the knockdown of AR triggered the inactivation of FGFRs, as indicated by the dephosphorylation of FGFRs and their downstream kinases, suggesting that AR regulates FGFR signalling, consistent with the findings of other studies.

Interestingly, we proved that FGFR2 signalling governs AR signalling as its upstream regulator [70, 71]. Treatment with either HA-P5 or AZD4547 (pan-FGFR inhibitor) or knockdown of FGFRs significantly decreased AR protein but not mRNA. Generally, the transcript and protein levels of a gene can be inconsistent during many physiological processes—for instance, miRNA and mRNA methylation. The results of this study indicated that FGFR signalling might regulate AR signalling via the regulation of m6A modification. We found that the decrease in FGFR signalling diminished the levels of AR protein in a YTHDF3-dependent manner. YTHDF3 belongs to the TYH family, which comprises YTHDF1, YTHDF2, and YTHDF3. They are m6A readers, according to recent research [72]. Generally, YTHDF3 forms a complex with YTHDF1 to facilitate transcription, while it forms another complex with YTHDF2 to degrade mRNA with m6A modification [73–76]. Our results are most likely an indicator that the YTHDF3/YTHDF1 complex improved the translation of AR mRNA. In this manner, the findings of our study support the notion that FGFR signalling and AR signalling form a cyclization to drive the formation of acne lesions.

According to the literature, IGF-1/PI3K/AKT signalling contributes to the development of acne lesions [77]. Increased AKT activation diminishes nuclear FoxO1 levels and reduces FoxO1-mediated suppression of AR [78, 79]. FoxO1 also regulates the expression of GATA6, a critical checkpoint regulating follicular keratinization [80]. In this study, we found the inactivation of AKT upon the inhibition of FGFRs. However, we did not observe significant changes in FoxO1 and GATA6 expression from the transcriptome analysis. Moreover, FoxO1 regulates the transcription of AR; however, AR mRNA levels did not change when exposed to P5 and AZD4547, indicating that the AKT-FoxO1-AR pathway might not be involved in the processes focused on in this study. Another AKT downstream pathway, MDM2/p53, contributes to acne pathogenesis [81, 82]. Notably, sebaceous gland differentiation is controlled by the p53/AR axis [83], and p53 inhibits the expression of AR [84]. However, the AKT-MDM2/p53 pathway might not be involved in the processes focused on in our study either because the expression of CDKN1A (p21), an indicator of p53 activation, is also stable when cells are exposed to HA-P5 and AZD4547. Moreover, p53 regulates AR through the induction of transcription, which is not affected by FGFR inhibitors. Our results suggested that FGFR signalling regulated AR signalling without the involvement of the FGFR- PI3K/AKT-FoxO1-AR and FGFR-PI3K/AKT-MDM2/p53-AR pathways.

Interestingly, unlike another FGFR inhibitor, AZD4547, HA-P5 did not trigger the elevation of AKR1C3. AKR1C3 encodes a pivotal enzyme that catalyzes testosterone [62, 85], which is one of the major culprits driving the formation and development of acne lesions [86, 87]. AZD4547 treatment, on the one hand, decreased FGFRs and AR signalling; however, on the other hand, it increased the formation of androgen, demonstrating the role of fence-sitters in acne therapy. The increased androgen induced by AZD4547 may be one of the potential reasons that HA-P5 exhibited better acne therapeutic activity than AZD4547 in vivo. The overexpression of AKR1C3 also contributed to the progression of multiple malignancies. These results indicated that HA-P5 is an optimized FGFR2 inhibitor for acne therapy.

We found that an HA polysaccharide conjugated and naturally derived oligopeptide, HA-P5, effectively remedies the symptoms of acne in vivo and in vitro. Mechanistically, HA-P5 inactivates FGFR2 to reduce the transcription of YTHDF3, which is crucial for the translation of AR. Consequently, sebum accumulation and cell proliferation are decreased to retard the formation and development of acne. Moreover, unlike AZD4547, HA-P5 does not trigger the overexpression of an obstacle gene for acne therapy, namely, AKR1C3, and exhibits stronger antiacne activities. Our findings highlighted HA-P5 as an optimized FGFR2 inhibitor for acne treatment and uncovered a potential mechanism by which FGFR2 regulates AR signalling.

### Supplementary Information

**Additional file 1: Figure S1.** Scheme of establishing the acne model on the male New Zealand rabbit ears. **Figure S2.** P5 and AZD4547 remedied acne lesions on the male New Zealand rabbit ears. **Figure S3.** The anti-acne activities of positive control and solvent controls on the male New Zealand rabbit ears.** (A) **Isotretinoin and solvent controls remedied acne on the ears of male New Zealand rabbits with smoother epidermis and thinner stratum corneum; **(B)** Isotretinoin and solvent controls diminished the hair follicles on the skin of rabbit ears; **(C)** Typical H&E staining images of the vertical sections of the hair follicles; **(D)** Quantification of the area of trichopore on the rabbit ears. One way ANOVA was employed for statistical analysis. ns in blue color refers to p>0.05 compared to the control group ** in blue color refers to p<0.01 compared to the control group, **** in blue color refers to p<0.0001 compared to the control group. #### in red color refers to p<0.0001 compared to the coal tar group.

## Data Availability

The datasets used in this study are available from the corresponding author upon reasonable request.

## Abbreviations

AR: Androgen receptor
AKR1C3: Aldo-keto reductase family 1 member C3
AFM: Atomic force microscope
BSA: Bovine serum albumin
bFGF: Basic fibroblast growth factor
BCA: Bicinchoninic acid
CCK8: Cell counting kit-8
DLS: Dynamic light scattering
DMSO: Dimethyl sulfoxide
DEPC: Diethyl pyrocarbonate
DMEM: Dulbecco’s modified eagle medium
DHT: Dihydrotestosterone
DEGs: Differentially expressed genes
DAPI: 4,6-Diamino-2-phenyl indole
ECL: Enhanced chemiluminescence
FGF2: Fibroblast growth factors 2
FGFRs: Fibroblast growth factor receptors
FGFR2: Fibroblast growth factor receptor 2
FGFs: Fibroblast growth factors
FOXO1: Forkhead box O1
FISH: Fluorescence in situ hybridization
FBS: Foetal bovine serum
GSEA: Gene set enrichment analysis
GATA6: GATA binding protein 6
HA: Hyaluronic acid
H&E: Haematoxylin/eosin
m6A: N6-methyladenosine
NES: Normalized enrich score
NTCC: National type culture collection
OCT: Optimal cutting temperature
PBS: Phosphate-buffered saline
PDGFB: Platelet derived growth factor subunit B
PPARG: Peroxisome proliferator activated receptor gamma
qPCR: Quantitative polymerase chain reaction ()
siRNA: Small interfering RNA
shRNA: Short hairpin RNA
SSC: Saline sodium citrate
TEM: Transmission electron microscopy
TG: Triglyceride
TSA: Tyramide signal amplification
TBST: Tris-buffered saline and tween 20
YTHDF3: YTH N6-methyladenosine RNA binding protein F3
