# Correction: Hyaluronic acid-FGF2-derived peptide bioconjugates for suppression of FGFR2 and AR simultaneously as an acne antagonist

**DOI:** 10.1186/s12951-024-02327-5

**Published:** 2024-02-19

**Authors:** Zijian Su, Yibo Zhang, Jieqiong Cao, Yuanmeng Sun, Yuling Cai, Bihui Zhang, Liu He, Zilei Zhang, Junye Xie, Qilin Meng, Lin Luo, Fu Li, Jingsheng Li, Jinting Zhang, Xiaojia Chen, An Hong

**Affiliations:** 1 Department of Cell Biology, College of Life Science and Technology, Jinan University; National Engineering Research Center of Genetic Medicine; Guangdong Provincial Key Laboratory of Bioengineering Medicine; Guangdong Provincial Biotechnology Drug & Engineering Technology Research Center, Jinan University, Guangzhou, Guangdong 510632 China; 2 The First Affiliated Hospital of Jinan University, Guangzhou, 510630 China

**Correction: Journal of Nanobiotechnology (2023) 21:55**

10.1186/s12951-023-01812-7

Following publication of the original article [1], the authors identified an incorrect statement about the origin of SZ95 human sebaceous gland cells in the cells and cell culture.

Therefore, in the cells and cell culture, the origin of SZ95 human sebaceous gland cells should be revised to “SZ95 human sebaceous gland cells were from Dr.Zouboulis’s Lab originally [2]”.

The original article [1] has been revised.
