# Supplementary material for: Hyaluronic acid-FGF2-derived peptide bioconjugates for suppression of FGFR2 and AR simultaneously as an acne antagonist
Source: J Nanobiotechnology. 2023 Feb 17;21:55. doi: 10.1186/s12951-023-01812-7 (PMC9938603; doi:10.1186/s12951-023-01812-7)

**Supplemental information**

**Figure S1.** Scheme of establishing the acne model on the male New Zealand rabbit ears.

**Figure S2.** P5 and AZD4547 remedied acne lesions on the male New Zealand rabbit ears.

**Figure S3.** The anti-acne activities of positive control and solvent controls on the male New Zealand rabbit ears. **(A)** Isotretinoin and solvent controls remedied acne on the ears of male New Zealand rabbits with smoother epidermis and thinner stratum corneum; **(B)** Isotretinoin and solvent controls diminished the hair follicles on the skin of rabbit ears; **(C)** Typical H&E staining images of the vertical sections of the hair follicles；**(D)** Quantification of the area of trichopore on the rabbit ears. One way ANOVA was employed for statistical analysis. ns in blue color refers to p>0.05 compared to the control group ** in blue color refers to p<0.01 compared to the control group, **** in blue color refers to p<0.0001 compared to the control group. #### in red color refers to p<0.0001 compared to the coal tar group.

Figure S1


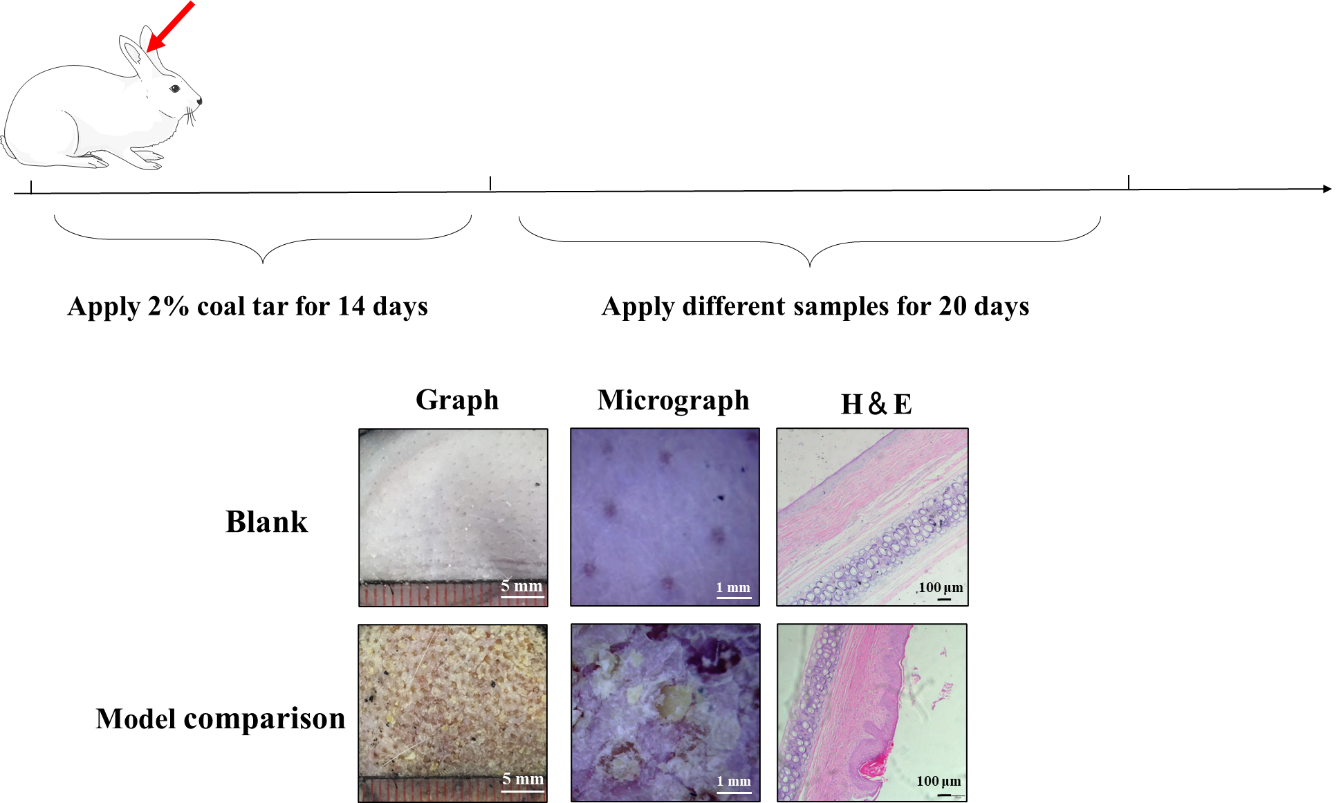


Figure S2


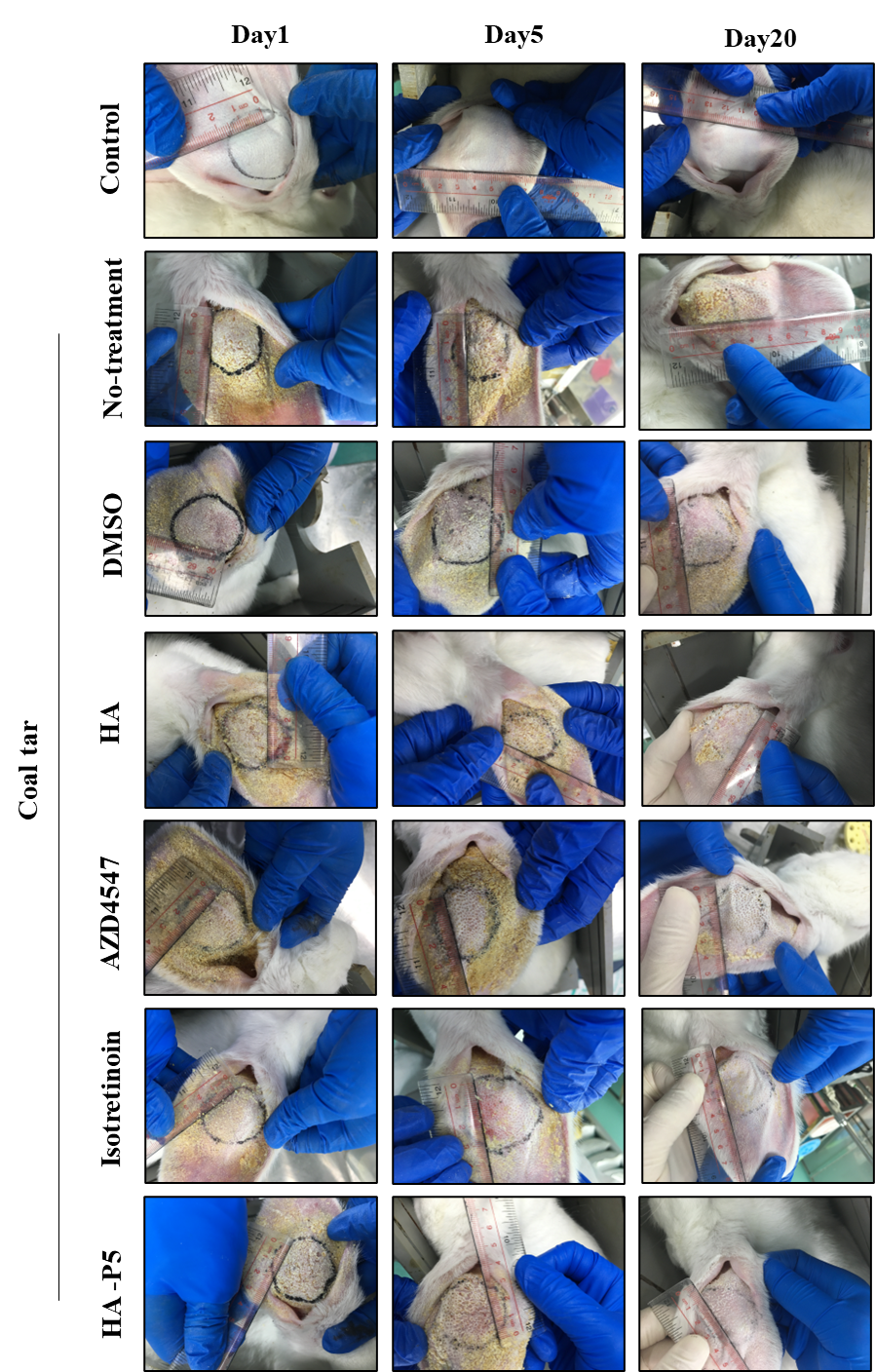


Figure S3


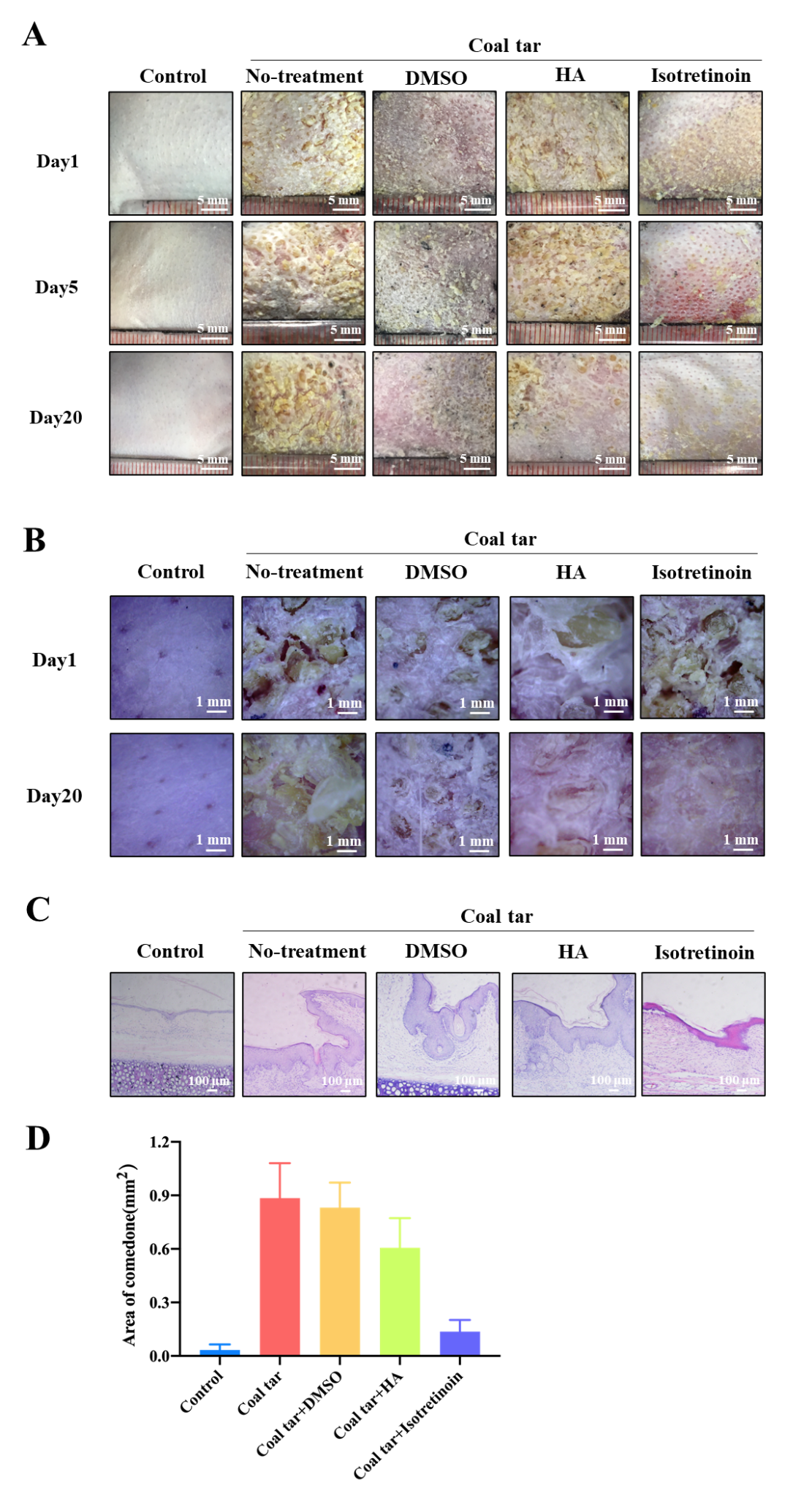

Supplement: Supplementary file 1 — Additional file 1: Figure S1. Scheme of establishing the acne model on the male New Zealand rabbit ears. Figure S2. P5 and AZD4547 remedied acne lesions on the male New Zealand rabbit ears. Figure S3. The anti-acne activities of positive control and solvent controls on the male New Zealand rabbit ears. (A) Isotretinoin and solvent controls remedied acne on the ears of male New Zealand rabbits with smoother epidermis and thinner stratum corneum; (B) Isotretinoin and solvent controls diminished the hair follicles on the skin of rabbit ears; (C) Typical H&E staining images of the vertical sections of the hair follicles; (D) Quantification of the area of trichopore on the rabbit ears. One way ANOVA was employed for statistical analysis. ns in blue color refers to p>0.05 compared to the control group ** in blue color refers to p<0.01 compared to the control group, **** in blue color refers to p<0.0001 compared to the control group. #### in red color refers to p<0.0001 compared to the coal tar group. [file 12951_2023_1812_MOESM1_ESM.docx]
